# The effects of social presence on cooperative trust with algorithms

**DOI:** 10.1038/s41598-023-44354-6

**Published:** 2023-10-14

**Authors:** Baptist Liefooghe, Ebelien Min, Henk Aarts

**Affiliations:** Utrecht University, Utrecht, The Netherlands

**Keywords:** Human behaviour, Computer science

## Abstract

Algorithms support many processes in modern society. Research using trust games frequently reports that people are less inclined to cooperate when believed to play against an algorithm. Trust is, however, malleable by contextual factors and social presence can increase the willingness to collaborate. We investigated whether situating cooperation with an algorithm in the presence of another person increases cooperative trust. Three groups of participants played a trust game against a pre-programmed algorithm in an online webhosted experiment. The first group was told they played against another person who was present online. The second group was told they played against an algorithm. The third group was told they played against an algorithm while another person was present online. More cooperative responses were observed in the first group compared to the second group. A difference in cooperation that replicates previous findings. In addition, cooperative trust dropped more over the course of the trust game when participants interacted with an algorithm in the absence another person compared to the other two groups. This latter finding suggests that social presence can mitigate distrust in interacting with an algorithm. We discuss the cognitive mechanisms that can mediate this effect.

## Introduction

Algorithms support many processes in today’s society, such as entertainment, service industry, administration, governance, transportation and health care^1–3^. The prolific use of algorithms raises the question how do we interact and cooperate with algorithms and, more generally, with artificial intelligence. A key determinant of this interaction is trust. Whereas trust depends on relatively objective features of an algorithm such as its performance, automation and transparency (for reviews, see^4–8,8,9^), trust in algorithms is also driven by the context that shapes the attitudes and beliefs that people hold towards algorithms^10–14^. Research on the strategic interaction or cooperation between humans and algorithms, such as in the context of economic decisions and social dilemma’s, frequently reports that humans are less cooperative and trustful when cooperating with an algorithm compared to another human^15–21^. In the present study, we investigate the role of a social context in changing trust when cooperating with an algorithm and test whether situating cooperation with an algorithm in the presence of another person promotes cooperative trust.

Cooperative behavior in a trust situation comprises four elements^22^: (a) Placing trust by a trustor allows a trustee to honor or abuse trust; (b) A trustor regrets placing trust if trust is abused, but benefits from honored trust; (c) A trustor voluntarily places resources in the hands of the trustee without formal safeguards and (d) There is a time-lag between placement of trust and the action of the trustee. Cooperative behavior is typically investigated in Trust Games, which offer a game-theoretic representation of a transaction that closely resembles the aforementioned criteria, see also^23–25^. A seminal example of a Trust Game is the prisoner’s dilemma (e.g.,^26^). In this game, a participant can decide to testify against an opponent (Defect) or remain silent (Cooperate). Remaining silent only pays off when the opponent also does so and being betrayed by the opponent while confessing leads to a higher penalty. Although some exceptions have been reported^19,20^, a common finding in different types of trust games is that less cooperative responses are made when the opponent is (or is believed to be) an algorithm compared to when the opponent is (or is believed to be) a real human^15–21^. Furthermore, prior commitments to cooperate in a trust game are more often broken off when playing against an algorithm^17^.

The reduced cooperative behavior that is observed when playing against an algorithm compared to a human opponent seems unaffected by the actual behavior of the algorithm. For instance^16^, conducted an online experiment in which participants played an iterated prisoner’s dilemma game. Participants were either led to believe they were coupled to another human player or an algorithm. On the very first round of the prisoners’ dilemma, the perceived opponent did not affect cooperation and the difference between a human and an algorithm opponent increased in the immediately following rounds. Importantly, over the course of the game the algorithm adapted to the responses of the participant such that it maximized cooperation. However, the mere knowledge that the opponent was an algorithm impeded a player’s willingness to cooperate.

Similarly^18^, conducted a lab-based experiment in which participants either introduced themselves face-to-face to a purported human opponent and then moved to their respective computers. When participants were led to believe they played against an algorithm, participants immediately sat in front of an assigned computer and engaged in the task. In both contexts, an algorithm was programmed as an opponent that could be highly cooperative or not. However, again more cooperative responses were made towards a human opponent, and this was not affected by the frequency of cooperative responses made by that opponent.

Cooperative behavior, however, is complex and can be influenced in a variety of ways that are not directly related to the nature of the cooperants. For instance, the social framing of the context in which the cooperation takes place influences the willingness to cooperate (e.g.,^27,28^). Eiser and Bhavnani^29^ observed more cooperative behavior in a Prisoners’ dilemma when the situation is framed as an international negotiation than when it is framed as a business transaction (see also^30–32^). Furthermore, cooperation can also be influenced by (un)intended social features of an opponent, such as emotional expression (see^34,35^ for reviews). Gallo and Dale^35^ reported that cooperation in the prisoners’ dilemma could be increased when the experimenter varied her tone of voice and facial expressions while delivering feedback during the game.

Previous research suggests that social context is also an important moderator in Human-AI interactions (see^36^ for a review). In line with the Social Presence Theory (e.g.,^37^), numerous interventions have been shown to facilitate pleasure and encourage people to experience interactions with computers as being social by adding physical features, such as pictures of faces, text, personalized greetings, or human audio and video^38–41^. For instance, the co-presence of an opponent in a computer game increases positive affect (e.g.,^42^) and creating the illusion of online social presence increases trust during online shopping^43^. Apparently, placing computers and algorithms in a social context changes the way people treat and respond to them.

Taken together, the findings discussed above indicate that trust when interacting with algorithms could be modulated by changing the social setting in which the interaction takes place. Here, we offer a proof of principle of this idea by testing whether cooperative trust towards an algorithm can be modulated when the trust game is embedded in a social context^43,44^. To this end, we conceptually replicated and extended the design used by Miwa and Terai^18^. Because the experiment was ran during the COVID pandemic, we used a set-up in which a virtual lab was used by means of the Microsoft Teams environment in which participants played a Trust Game. In the Other-Person-as-Opponent condition, participants were led to believe that they played the trust game against another person that was present online during the experiment and to which they were introduced to face-to-face by means of a webcam. In the Algorithm-as-Opponent condition participants were informed that they played against an algorithm during the experiment and no other person was present online in the game context. Finally, in the Algorithm-as-Opponent-in-Social-Context condition participants were told that they played against an algorithm, but another person was present online during the experiment to which they were also introduced to face-to-face.

**Figure 1:**
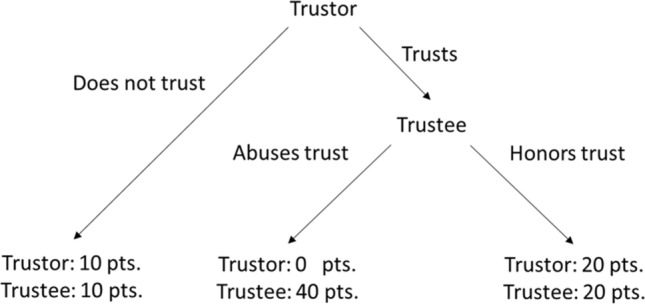
Structure and pay-off scheme of the trust game.

We used an iterative trust game that matches the definition of^22^ and additionally enables to investigate separately the two roles that can be endorsed in a trust situation, namely trustor or trustee. During this iterative trust game^45,46^, a participant decides to trust an opponent or not on a first round. This decision is followed by a message indicating that the opponent honors or abuses the trust granted by the participant. As can be seen in Fig. 1, all choices made by the players lead to different rewards or losses. In the next game round, the roles are reversed, and the opponent granted trust (or not) and the player could decide to honor this trust or not. Participants thus made two cooperative responses in succession. First, they indicate whether they trust the opponent and want to play one round of the game (trust granting). Second, they decide either to abuse or honor the trust given by the opponent (trust honoring). This additional response makes it possible to further investigate the observation that players are more inclined to break prior commitments or less honor trust that was granted to them by an algorithm^17^. Furthermore, the decision to grant and to honor trust in a game round was repeated across 64 game rounds. Earlier findings suggest that the bias against algorithms increases over time^16^. As such, we could test whether we could replicate such increase in bias and if social presence can mitigate this effect. In addition, the frequency of abusive responses emitted by the opponent could either be high (75%) or low (25%) in two separate game sessions. As such, we could again test whether biases towards an opponent were affected by the actual behavior of the opponent^16,18^.

Building on previous research, we expected that participants would show more cooperative responses in the Other-Person-as-Opponent condition than in the Algorithm-as-Opponent condition (see also^18,20–24^). In addition, this difference may increase over the course of the game^16^ and remain unaffected by the frequency of abusive responses made by the opponent^18^. The novel condition in the present study is the Algorithm-as-Opponent-in-Social-Context condition in which cooperation takes place in a social context, while disclosing that the opponent is an algorithm. As such, we could estimate the influence of social presence on trust in the cooperation with an algorithm. On the one hand, if the distrust against an algorithm is robust as was suggested before^16^, the influence of social presence may be minimal. As such, the pattern of cooperative responses (frequency, stability over the course of the game), should be similar to the pattern of cooperative responses in the Algorithm-as-Opponent condition because in both conditions the purported opponent is an algorithm. On the other hand, if in line with Social Presence Theory, the influence of the social context is substantial, then the pattern of cooperative responses (frequency, stability over the course of the game) in the Algorithm-as-Opponent-in-Social-Context should be similar to the pattern in the Other-Person-as-Opponent condition. Note that previous research used the prisoners’ dilemma, while our task differs and required two explicit responses: trust granting and trust honoring. As this distinction is new, we made no specific predictions about how our different conditions could affect these specific responses.

## Method

### Participants

Hundred-and-one participants were enrolled in the experiment either voluntarily or for course credits. Participants were recruited from the social network of the second author and by means of Sona Systems (https://www.sona-systems.com/) and randomly assigned to one of the three between-subjects conditions of the experiment: the Other-Person-as-Opponent condition (*n* = 32), the Algorithm-as-Opponent condition (*n* = 34), and the Algorithm-as-Opponent-in-Social-Context condition (*n* = 35). In view of the elaborateness of the experimental sessions we opted for a convenience sample. As mentioned in the Introduction, the present study was conceptually similar to the study of^18^, as it used a between-subjects comparison of conditions in which participants were introduced face-to-face with an opponent or other person. The main difference was that we used an online setting with a webcam instead of physical interactions because we ran our study during the COVID pandemic. Miwa and Terai^18^ used samples of around 20 participants in each condition. The average effect size of the difference in cooperation when the purported opponent was a human or an algorithm was a Cohens’ *d* of 0.60. This is above a medium size effect of *d* = 0.5^47^. However, because our design also included a within-subjects manipulation, namely the frequency of abusive responses of the opponent, a minimum of 32 participants per group was set, which offers a power of 0.8 for detecting a medium size effect of *d* = 0.5^47^, in each condition.

Due to an error, the age of the participants was not registered and the gender of the sample was only partially available, which consisted at least of 28 men and 40 women. The experimental protocol was approved by the Ethical Review Board of the Faculty of Social and Behavioral Sciences of Utrecht University (21-2049). The experiment was performed in accordance with relevant guidelines/regulations in line with the Declaration of Helsinki. Informed consent was obtained for all participants.

### Trust game

The trust game was programmed and webhosted in Gorilla^48^. In each round of the trust game, a player first decided to trust an opponent or not, who in turn could honor or abuse this trust. As can be seen in Fig. 1, all the choices made by the players lead to different rewards or losses. On the next round, the roles were reversed, and the opponent granted trust (or not) and the player could decide to honor this trust or not. With this second response we further investigated the observation that players are more inclined to break prior commitments or less honor trust that was granted to them by an algorithm^17^. The frequency of cooperative responses emitted by the algorithm could either be high (75%) or low (25%) in two separate game sessions. Note that, these frequencies were determined at the level of a session and on each round of the game incertitude remained to whether a participant would be abused or not by the opponent. As such, we could investigate how possible biases towards the algorithm were affected by the cooperativeness of the algorithm^16,18^.

At the start of the first round of the game, participants were presented with the question: “*Do you trust your opponent?*” in the middle of the screen. They responded to this question by means of the ‘v’ (yes) and ‘n’ (no) keys of their keyboard. If participant pressed ‘no’, they proceeded to the end of the round. If participants pressed ‘yes’, their trust could either be honored or abused by the opponent. In the first case, the message “*The opponent honors your trust! You both win 20 points!*” appeared in the center of the screen and in the second case the message: “*The opponent abuses your trust! You win 0 points! The opponent wins 40 points!*”. Participants could then press the spacebar to proceed. At the end of the round, the total number of points earned so far was mentioned: *“You have XXX points. The opponent has XXX points.”* The number of points earned had no consequence for the participants.

The opponent started the next round and the participants either received the message “*The opponent does not trust you!*” or “*The opponent trusts you! Do you honor the opponent's trust?”*. In the first case, the participants proceeded to the end of the round by pressing the spacebar. In the second case, participants responded by pressing ‘yes’ or ‘no’, before proceeding to the end of the round, at which point a summary of the points earned so far was presented. The next round was again opened by the participant. Participants thus alternated systematically between the role of trustor and trustee. The dependent variables were the frequency of trust-granting responses and the frequency of task-honoring responses.

### Procedure

In the Other-Person-as-Opponent and Algorithm-as-Opponent-in-Social-Context condition participants were invited to a video call in Microsoft Teams with the experimenter (female, mid-twenties, Caucasian). The video call started with a short welcome word introducing the experimenter and the experiment. Following this welcome word, a link was sent via the chat function of Teams. By clicking this link, they could start the experiment on their own laptop or PC. Participant were asked to maximize the screen of the Gorilla environment, such that they could no longer see the Teams environment and the experimenter. Participants then received more instructions about the trust game and their opponent. In each condition the experimenter was introduced (name, e-mail). In the Other-Person-as-Opponent condition participants were informed to play a trust game against the experimenter. In the Algorithm-as-Opponent-in-Social-Context condition, participants were told that they would play against an algorithm that was programmed by the experimenter, who would also be present during the experiment. In the Algorithm-as-Opponent condition, the introduction text was identical as in the Algorithm-as-Opponent-in-Social-Context condition. However, participants were directed to the experiment via a link in the Sona recruitment system and no Teams environment or video call was used. In all three conditions, the instructions were followed by an informed consent, whereafter the trust game started.

Following the instructions, the trust game started. Two blocks of 64 rounds were presented: the 75%-trust-honoring block and the 25%-trust-honoring block. In each block, participants systematically alternated between the roles of trustor (i.e., granting trust) and trustee (i.e., honoring trust). They started always as trustor in the first round. The same algorithm was used in each condition. In the 75%-trust-honoring block, the algorithm was programmed to make trust honoring responses on 75% of the rounds. In the 25%-trust-honoring block, the algorithm was programmed to make trust honoring responses on 25% of the rounds. In both blocks, these responses were randomly dispersed per participant. When the player was the trustee, the algorithm granted trust in 75% of the rounds in each block, again randomly dispersed per participant.

### Data processing and analyses

Data were processed an analyzed in R. The number of cooperative responses (trust honoring, trust granting) were considered as main dependent variables. General linear mixed models were used as implemented in the package ‘lme4′^49^. The Block Type (75% vs. 25% trust honoring) and the Condition (Other Person-as-Opponent, Algorithm-as-Opponent, Algorithm-as-Opponent-in-Social-Context) were fixed effects and effect coded.

The Game Round was added as a numerical predictor (64 rounds). After visual inspection of the data, we noticed a steeper drop in cooperative responses in the first rounds of the game. In order to account for this non-linear trend, round numbers were first log-transformed and subsequently centered^50^. A random intercept was estimated per participant. Finally, individual differences in the effect of Block Type and Game Round, as well as their interaction were also included in the model. We thus used the maximal random-effect structure^51^. The reported *p-*values for the fixed effects are based on a Type III ANOVA using a ${\chi }^{2}$-distribution. Estimated marginal means and follow-up *z*-ratio tests were calculated with the package ‘emmeans’^52^. Multiple comparisons were corrected for Type I error inflation by using Tukey’s method.

## Results

### Trust granting

The main effect of Condition Type was significant, ${\chi }^{2}$(2) = 7.74, *p* = 0.02. The probability of trust granting was higher in the Other-Person-as-Opponent condition (*M* = 0.43; *SE* = 0.06) compared to the Algorithm-as-Opponent condition (*M* = 0.23; *SE* = 0.04), *z* = 2.75, *p* = 0.02. The probability of trust granting in the Algorithm-as-Opponent-in-Social-Context condition (*M* = 0.30; *SE* = 0.05) did not differ significantly from the Other-Person-as-Opponent condition, *z* = 1.79, *p* = 0.17, and the Algorithm-as-Opponent condition, *z* < 1. The main effect of Block Type was significant, ${\chi }^{2}$(2) = 43.69, *p* < 0.001. The probability of trust granting in the 75%-trust-honoring condition (*M* = 0.42; *SE* = 0.04) was higher than in the 25%-trust-honoring condition (*M* = 0.22; *SE* = 0.02). Finally, the probability of trust granting decreased with the Game Round, ${\chi }^{2}$(1) = 84.65, *p* < 0.001.

**Figure 2:**
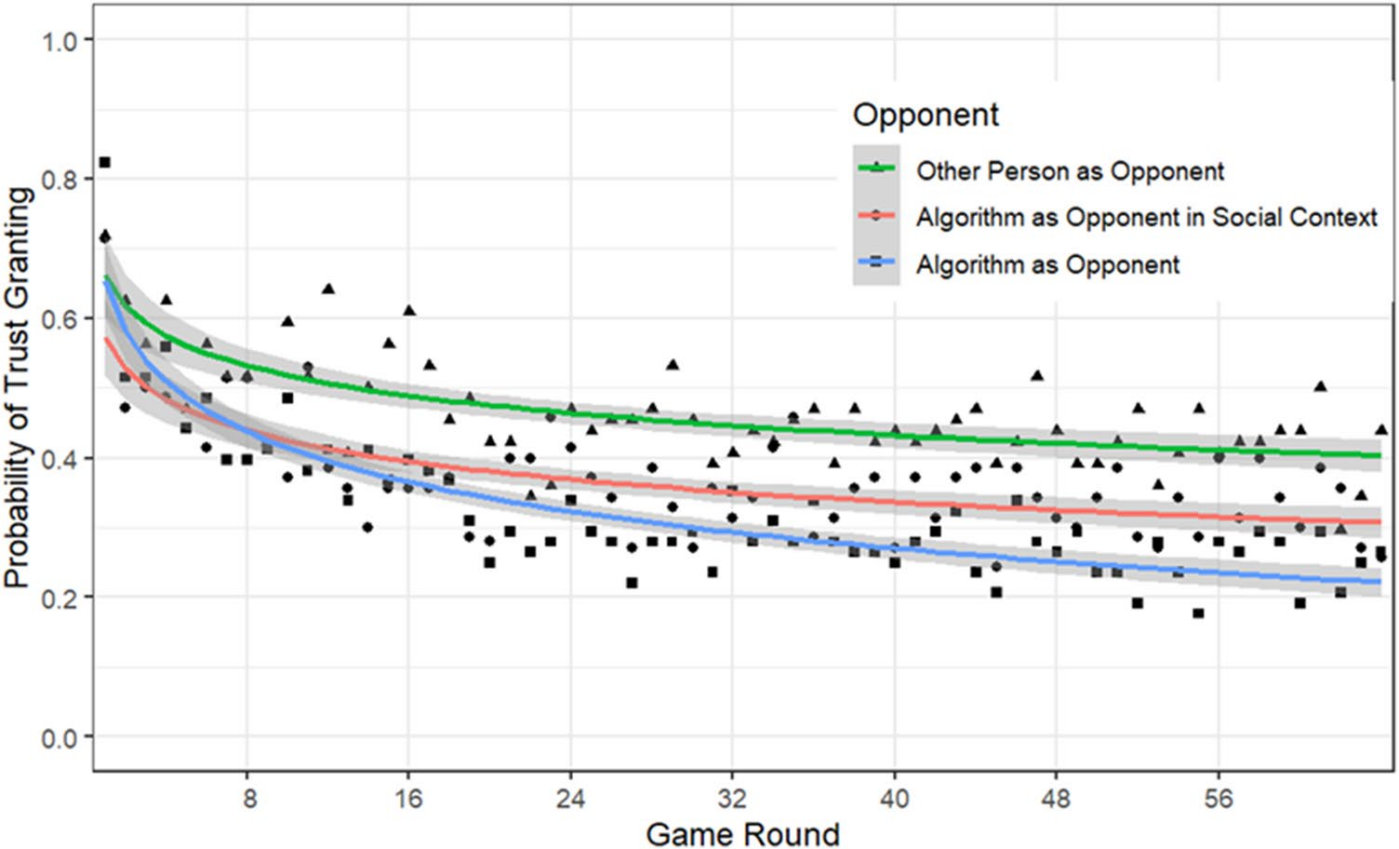
Probability of trust granting as a function of opponent and game round.

The interaction between Condition Type and the Game Round was significant, ${\chi }^{2}$(2) = 8.25, *p* = 0.02 (see Fig. 2). These and further interactions with Game Round were decomposed by comparing the slopes by which trust granting decreased as a function of the Game Round in the different conditions of interest. This decrease was significantly steeper in the Algorithm-as-Opponent condition compared to the Other-Person-as-Opponent condition, *z* = 2.65, *p* = 0.02, and not significantly steeper compared to the Algorithm-as-Opponent-in-Social-Context condition, *z* = 2.29, *p* = 0.06. The difference in decrease between the Other-Person-as-Opponent and the Algorithm-as-Opponent-in-Social-Context condition was not significant, *z* < 1.

**Figure 3:**
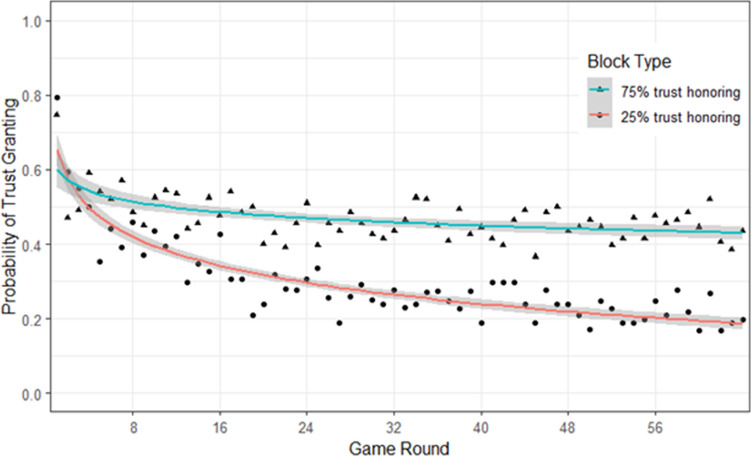
Probability of trust granting as a function of block type.

The interaction between Block Type and the Game Round was significant, ${\chi }^{2}$(2) = 22.35, *p* < 0.001 (see Fig. 3). The decrease in trust granting was significantly steeper in the 25%-trust-honoring condition compared to the 75%-trust-honoring condition. Finally, the interaction between Block Type and Condition, ${\chi }^{2}$(2) < 1, and the three-way interaction, ${\chi }^{2}$(2) = 2.09, *p* = 0.36, were not significant.

### Trust honoring

The main effect of Condition Type was not significant, ${\chi }^{2}$(2) = 3.21, *p* = 0.20: Other-Person-as-Opponent condition: *M* = 0.34, *SE* = 0.06; Algorithm-as-Opponent condition: *M* = 0.20, *SE* = 0.05; Algorithm-as-Opponent-in-Social-Context condition: *M* = 0.23, *SE* = 0.05. The main effect of Block Type was significant, ${\chi }^{2}$(2) = 31.47, *p* < 0.001. The probability of trust honoring in the 75%-trust-honoring condition (*M* = 0.35; *SE* = 0.05) was higher than in the 25%-trust-honoring condition (*M* = 0.18; *SE* = 0.02). Finally, the probability of trust honoring decreased with the Game Round, ${\chi }^{2}$(1) = 62.83, *p* < 0.001.

**Figure 4:**
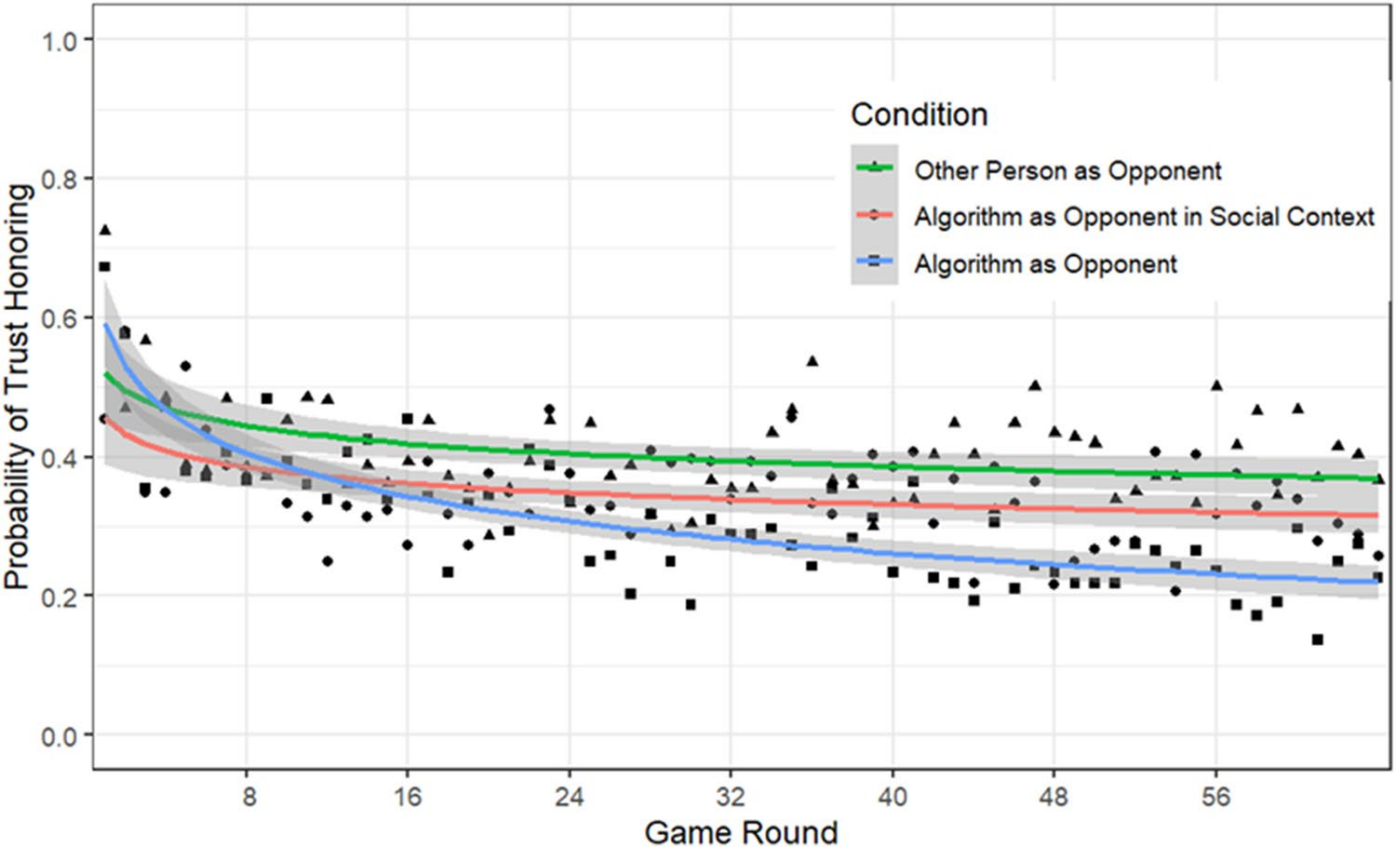
Probability of trust honoring as a function of opponent and game round.

The interaction between Condition Type and Game Round was significant, ${\chi }^{2}$(2) = 10.38, *p* = 0.006 (see Fig. 4). The decrease in trust honoring was significantly steeper in the Algorithm-as-Opponent condition compared to the Other-Person-as-Opponent condition, *z* = 3.07, *p* = 0.006, and compared to the Algorithm-as-Opponent-in-Social-Context condition, *z* = 2.39, *p* = 0.045. The difference in slopes between the Other-Person-as-Opponent and the Algorithm-as-Opponent-in-Social-Context condition was not significant, *z* < 1. The interaction between Game Round and Block Type, ${\chi }^{2}$(2) = 1.49, *p* = 0.22, and between Block Type and Opponent, ${\chi }^{2}$(2) = 2.74, *p* = 0.05 were both not significant. The three-way interaction was also not significant, ${\chi }^{2}$(2) < 1.

### Additional analyses

The above analyses suggest that social presence mitigates the decrease in cooperative responses over the course of the trust game. However, contrasts testing these trends were sometimes near the conventional 0.05 significance criterion and when decomposing the main effect of Condition Type for the trust-granting responses, only the difference between the Algorithm-as-Opponent condition and the Other-Person-as-Opponent condition was significant. Our findings may thus be limited by a lack of power resulting from the convenience sample we used. We therefore present three additional sets of analysis to investigate how likely our analyses can lead to correct inferences (see also^53^ for a related approach). Each analysis aims to increase the robustness of our findings. We only highlight the relevant effects. Full analyses and corresponding scripts were added to the supplementary materials.

#### Combining both types of responses in the same analysis

Trust granting and trust honoring are two types of cooperative responses, the increase of which indicate that a participant is more willing to cooperate and trust the opponent. As such both measures can be combined, leading to more observations per participant when focusing on the effects of interest, which increases the power of the test (see also^54^). When entering both dependent variables in the same analysis and adding Response Type as an independent variable to our model, the main effect of Condition Type was significant, ${\chi }^{2}$(2) = 6.10, *p* = 0.047. However, the difference in cooperative responses between the Algorithm-as-Opponent condition compared to the Other-Person-as-Opponent condition was not significant, *z* < 1. The interaction between Condition Type and Opponent was significant, ${\chi }^{2}$(2) = 15.81, *p* < 0.0001. The decrease in the probability of a cooperative response was significantly steeper in the Algorithm-as-Opponent condition compared to the Other-Person-as-Opponent condition, *z* = 3.49, *p* = 0.006, and compared to the Algorithm-as-Opponent-in-Social-Context condition, *z* = 2.63, *p* = 0.002.

#### Simplifying the random structure of the model

The mixed linear effects models in our main analyses used a maximal random-effect structure^51^, which can lead to a significant loss in power^55,56^. Accordingly, we refitted the models of our main analyses by only estimating random intercepts. For the trust-granting responses, the main effect of Condition Type was significant, ${\chi }^{2}$(2) = 7.75, *p* = 0.02. However, the difference in cooperative responses between the Algorithm-as-Opponent condition compared to the Other-Person-as-Opponent condition was not significant, *z* < 1. The interaction between Condition Type and Game Round was significant, ${\chi }^{2}$(2) = 25.14, *p* < 0.001. The decrease in trust granting was significantly steeper in the Algorithm-as-Opponent condition compared to the Other-Person-as-Opponent condition, *z* = 4.68, *p* = 0.001, and compared to the Algorithm-as-Opponent-in-Social-Context condition, *z* = 3.93, *p* < 0.001. For the trust-honoring responses, the main effect of Condition Type was not significant, ${\chi }^{2}$(2) = 3.11, *p* = 0.21. The interaction between Condition Type and Game Round was significant, ${\chi }^{2}$(2) = 8.66, *p* = 0.003. The decrease in trust honoring was significantly steeper in the Algorithm-as-Opponent condition compared to the Other-Person-as-Opponent condition, *z* = 5.11, *p* < 0.001, and compared to the Algorithm-as-Opponent-in-Social-Context condition, *z* = 4.49, *p* < 0.001.

#### Centered treatment contrasts

To target our research question more directly, treatment contrasts can also be used in which the Algorithm-as-Opponent condition is considered as the base level. Centered treatment contrasts were assigned to the categorical predictors^55,56^ and the maximum random structure was again used^51^. For the trust-granting responses, the contrast between the Algorithm-as-Opponent condition and the Other-Person-as-Opponent condition was significant, *z* = 2.75, *p* = 0.006. The contrast between the Algorithm-as-Opponent condition and the Other-Person-as-Opponent-in-Social-Context condition was not significant, *z* = 1.00, *p* = 0.32. The decrease in trust granting was significantly steeper in the Algorithm-as-Opponent condition compared to the Other-Person-as-Opponent condition, *z* = 2.71, *p* = 0.006, and compared to the Algorithm-as-Opponent-in-Social-Context condition, *z* = 2.26, *p* = 0.02. For the trust-honoring responses, the contrast between the Algorithm-as-Opponent condition and the Other-Person-as-Opponent condition and the contrast between the Algorithm-as-Opponent condition and the Other-Person-as-Opponent-in-Social-Context condition were not significant, *z* = 1.73, *p* = 0.08, and *z* < 1, respectively. The decrease in trust honoring was significantly steeper in the Algorithm-as-Opponent condition compared to the Other-Person-as-Opponent condition, *z* = 3.11, *p* = 0.002, and compared to the Algorithm-as-Opponent-in-Social-Context condition, *z* = 2.37, *p* = 0.02.

## Discussion

The different analyses presented in the previous section can be summarized as follows. First, more trust was granted to the opponent when believed to play against a human as compared to when being aware to play against an algorithm in the absence of an online person during the experiment. This difference echoes previous research that observed reduced cooperative trust towards algorithms^15–21^. Second, when being aware to play against the algorithm and a person was present online, the probability of trust granting did not differ significantly from the other two conditions. Third, over the course of the game, the probability of trust granting decreased. This decrease was more pronounced when no online person was present, compared to the other two conditions. For the sake of transparency, we mention again that in our main analysis the relevant contrast between the decrease in trust granting in the Algorithm-as-Opponent and the Algorithm-as-Opponent-in-Social-Context condition was not significant, *p* = 0.06. In all other additional analyses, the contrast was significant following a 0.05 criterion. Taken together, these findings suggest that social presence can mitigate the effect of distrust over the course of a trust game.

The probability of the trust-honoring response did not vary as a function of the purported opponent. The probability of trust honoring decreased over the course of the game. This decrease was more pronounced when no other online person was present, compared to the other two conditions. This finding also suggests that social presence can attenuate distrust towards an algorithm, albeit over the course of the game. Furthermore, the results of the trust-honoring responses are in line with previous research suggesting that participants are less inclined to honor trust granted by an algorithm^17^.

Our findings are in line with previous demonstrations in social cognition and behavior, showing that cooperation and trust between humans are malleable by the social context (e.g.,^27,28,30–34,44^). The present findings add to this literature by emphasizing the importance of social context in Human-AI interactions, and specifically the role of social presence in cooperative trust towards an algorithm. Our findings corroborate with the work of^43^, which was inspired by the Social Presence Theory^37^, who observed that creating the illusion of online social presence increases trust during online shopping. Yet, we remain cautious in our conclusion: Our findings suggest that social presence counteracts the decrease in cooperation over the course of a trust game. This is different from saying that social presence actually ‘boosts’ cooperation.

Our demonstration is only a proof of principle and the social cognitive mechanisms underlying the effect of social presence on cooperative trust in AI need to be further refined. According to intention-based approaches of cooperation^59^, a key element of cooperation are the intentions that opponents attribute to one another. Such attribution of intentional states or mentalizing^60^ helps us explain the behavior of other agents by attributing to them mental states of various sorts: beliefs, desires, and so on. In the context of a trust game, player A may reciprocate the risky decision to trust made by player B because the decision of the player B signals the intention to trust player A (i.e., a reciprocal-trust relationship). Evidence of such intention-based reciprocity, typically, comes from studies observing more cooperative responses in situations in which an opponent’s action was chosen intentionally compared to the situation in which the opponent’s action was implemented by a random device or was the only option available^61–63^. Following this view, cooperation thus depends on adopting an “intentional stance”^64^ towards an opponent. Although algorithms are usually not considered in terms of agency, there is some evidence suggesting that trust in algorithmic applications (e.g., robots, intelligent navigation systems) depends on the degree to which these applications are perceived to be human or possess intentionality^5,16^.

Within the account addressed above, the online presence of another person may have installed a social context that caused participants to perceive the algorithm as more intentional. Such “social transference” effect is common in a social interaction context where people spontaneously infer traits, goals and abilities from the behavior of interaction partners^65,66^ and attribute them to others who are also involved in the interaction^67,68^. The mere knowledge that the algorithm was programmed by the person present during the game, may also have strengthened the perception of the algorithm being intentional as the person that was present online (see also^69^ for higher social preferences for algorithms that are known to be related to a human).

Of course, alternative accounts need to be considered that can also explain the difference in trust when cooperating with a human or with an algorithm. For instance, human factors research suggests that people commonly expect algorithms to be more consistent than humans^70^. An algorithm could thus be expected to adapt to a lesser degree as an opponent (i.e., using a tit-for-tat strategy). A such, it may be assumed by a player that the algorithm will unlikely change strategy and retaliate when being less cooperative or even abuse the trust of that algorithm.

The difference in cooperation towards humans and algorithms may also follow from a more general feeling of *algorithm aversion*^71^, that is independent about one’s inferences about the workings of that algorithm (intentionality, consistency). For instance, it has been observed across different forecast domains that people often prefer to endorse forecasts of other humans rather than use algorithms, even when the latter are more accurate (e.g.,^72–74^). Such explanation fits with our observation that the difference in cooperation towards humans and algorithms was not affected by the extent to which the algorithm produced cooperative responses. A finding which is in line with previous studies^16,18^. Interestingly, a recent study by Liefooghe et al.^75^ suggests that even at the early of stage of social impression formation people tend to distrust artificial intelligence. These authors presented pictures of real faces and noted that merely labelling these faces as being artificial resulted in these faces to be rated as less trustworthy. This finding suggests that distrust against algorithms in the context of strategic cooperation may result from a prejudice that precedes our actual experience with these algorithms.

In a similar vein, the effect of the presence of others on people’s social mind can also be explained without calling upon the social attribution of perceived intentionality. The presence of others has been linked to the phenomenon of the audience effect, which causes people to become more self-aware^76,77^ and to search for cues of approval or disapproval from others^78,79^. This effect has been reported in different ecological settings, including face-to-face interactions as well as online visible and invisible interactions. The enhanced self-focused attention leads to the consideration of social rules and norms and encourages people to change behavior when actions are incongruent with these norms. According to this notion, participants showed more trust towards the algorithm in the presence of the person who programmed it, because they considered this behavior in line with leading norms about cooperation in the social context at hand.

To conclude, we observed that people trust algorithms more when interacting with them in the presence of others. Although this finding was predicted on the basis of Social Presence Theory^37^, future research will need to pinpoint the cognitive mechanisms underlying this effect. At the same, our results indicate a functional relationship between social presence and trust, which can be used to develop future interventions, even if these cognitive mechanisms are still unclear (see^80^ for a similar point in applied psychology). The observation that cooperation towards an algorithm is to a certain degree affected by social presence and not by the behavior of the algorithm offers new insights about these mechanisms and spurs new research directions.

## Data Availability

Data and analysis scripts are available at https://osf.io/2n7cx/.
